# TransRender: a transformer-based boundary rendering segmentation network for stroke lesions

**DOI:** 10.3389/fnins.2023.1259677

**Published:** 2023-10-12

**Authors:** Zelin Wu, Xueying Zhang, Fenglian Li, Suzhe Wang, Jiaying Li

**Affiliations:** ^1^College of Electronic Information and Optical Engineering, Taiyuan University of Technology, Taiyuan, China; ^2^The First Clinical Medical College, Shanxi Medical University, Taiyuan, China

**Keywords:** transformer, deep learning, stroke, segmentation, boundary

## Abstract

Vision transformer architectures attract widespread interest due to their robust representation capabilities of global features. Transformer-based methods as the encoder achieve superior performance compared to convolutional neural networks and other popular networks in many segmentation tasks for medical images. Due to the complex structure of the brain and the approximate grayscale of healthy tissue and lesions, lesion segmentation suffers from over-smooth boundaries or inaccurate segmentation. Existing methods, including the transformer, utilize stacked convolutional layers as the decoder to uniformly treat each pixel as a grid, which is convenient for feature computation. However, they often neglect the high-frequency features of the boundary and focus excessively on the region features. We propose an effective method for lesion boundary rendering called TransRender, which adaptively selects a series of important points to compute the boundary features in a point-based rendering way. The transformer-based method is selected to capture global information during the encoding stage. Several renders efficiently map the encoded features of different levels to the original spatial resolution by combining global and local features. Furthermore, the point-based function is employed to supervise the render module generating points, so that TransRender can continuously refine the uncertainty region. We conducted substantial experiments on different stroke lesion segmentation datasets to prove the efficiency of TransRender. Several evaluation metrics illustrate that our method can automatically segment the stroke lesion with relatively high accuracy and low calculation complexity.

## 1. Introduction

Reliable segmentation is the cornerstone for identifying disease types and making treatment strategies, and it plays an indispensable role in assisted therapy and intelligent healthcare (Tajbakhsh et al., 2020). Deep learning-based methods attract enormous research interest in various segmentation tasks, such as stroke lesion segmentation (GBD 2016 lifetime risk of stroke collaborators, 2018; Wu Z. et al., 2023), skin lesion segmentation (Yuan et al., 2017; Khattar and Kaur, 2022), and brain tumor segmentation (Pereira et al., 2016; Huang P. et al., 2022). Ischemic stroke is a series of sudden neurological deficits caused by localized cerebral ischemia and permanent infarction, and it has become a major cause of injury and even death (Matsuo et al., 2017). For the detection and treatment of stroke, magnetic resonance imaging (MRI) has become an indispensable method with the advantage of high resolution. Deep learning-based techniques produce rapid and accurate lesion segmentation that assists physicians in making timely medical decisions (Nielsen et al., 2018). In the last decade, convolutional neural networks (CNN) have grown popular for researchers in the image processing field due to their success at extracting feature representations (Wu J. et al., 2023). U-Net (Ronneberger et al., 2015) is a popular encoder-decoder symmetric structure that achieves great success for various 2D segmentation tasks. Many of the proposed methods (Milletari et al., 2016; Schlemper et al., 2019; Zhou Y. et al., 2021) are improved based on U-Net, providing spatial information, semantic information, and more. However, CNN-based methods are intractable for establishing long-distance features because of the limitations of their inherent structure.

In the last few years, transformer (Vaswani et al., 2017), which originated in the field of natural language processing (NLP), has shown great potential in a series of visual tasks. The vision transformer (Dosovitskiy et al., 2021; Wang et al., 2021; Chen et al., 2022) is applied directly from NLP to image classification task and outperforms the CNN-based methods. Transformer and its derived methods demonstrate impressive achievements in a variety of visual tasks. The pure transformer is not appropriate, and the structure of hybrid CNN-transformer methods becomes the model of choice in medical image analysis (He et al., 2022). TransUNet (Chen et al., 2021), the first hybrid architecture in medical field, extracts the global features of medical images through transformer layers. For the organs segmentation, TransUNet realizes excellent results that outperform existing CNN-based methods. In contrast to the cascade structure, TransFuse (Zhang et al., 2021a) utilizes both CNN and transformer in a parallel connection. The above-mentioned methods refine the feature representation of the encoder from different perspectives, while for the decoder they employ the traditional convolutional upsampling method. It's undeniable that the long range modeling capability of transformer is very powerful.

**Figure 1:**
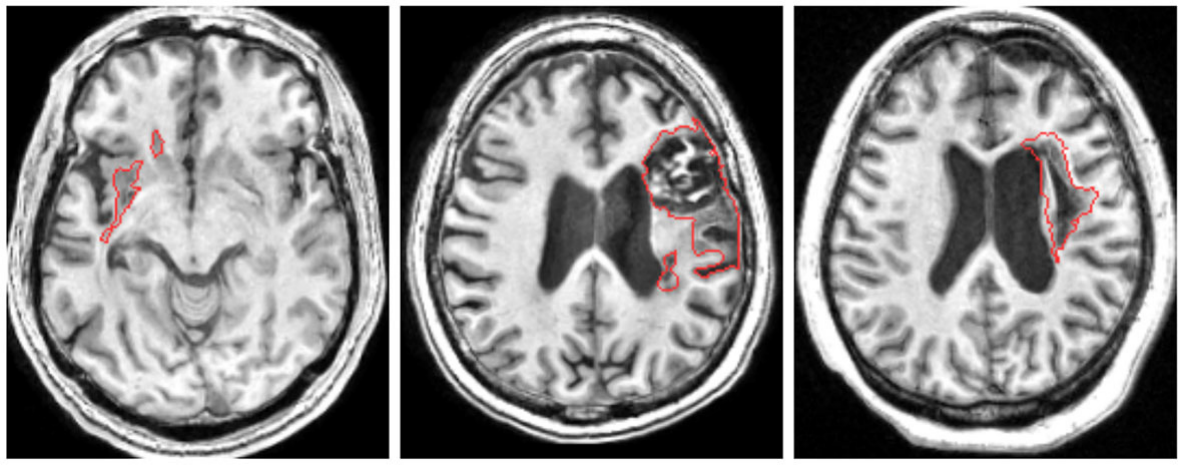
Stroke lesions are distributed in different locations and have extremely irregular sizes and shapes. Furthermore, the similarity of the lesion to the surrounding healthy tissue further increases the difficulty of segmentation.

Unfortunately, stroke lesion segmentation still faces enormous challenges, as shown in Figure 1. and the difficulty of identifying lesion boundaries. We can see that the location of the lesions are different due to the individual differences of patients and their lifestyle habits. The uncertain location of occurrence and the complex brain structure cause the shape of the lesion is extremely irregular. Furthermore, the statistical features of focal tissue are not significantly different from those of healthy tissue, leading to challenging segmentation of irregular lesion boundaries.

Most of the existing methods tend to treat all feature representations of the lesion region uniformly in a regular grid way, ignoring the high-frequency information at the boundaries, which makes the segmentation of the lesion boundary more difficult.

To ameliorate these difficulties, we propose a point-based boundary segmentation method, TransRender, which comprises the transformer as the encoder and the render-based module as the decoder. The transformer-based encoder constructs global features of the input image sequence at several scales. The render-based decoder utilizes a subdivision strategy that adaptively selects an uncertain set of points to recompute the original segmentation. Furthermore, the render module leverages both CNN and transformer features to recover the resolution of the segmentation results, which enriches the local-global features of the deep semantic information. To illustrate the validity of the TransRender, we implement the comparative experiments using different stroke lesion datasets. The experimental results from these datasets suggest that TransRender achieves excellent performance in the lesion segmentation task.

To summarize, our main contributions are as follows:

1. We construct a boundary-related network structure for stroke lesion segmentation, called TransRender, by adopting both the multi-scale transformer to build long-distance dependency and render-based decoder to compute the original recovery images.
2. We propose a render-based decoder that is trained to predict uncertain points, allowing the decoder to fine-tune the lesion boundaries.
3. We design multi-level point-to-point supervision to optimize the point selection strategy. The comprehensive experiments are conducted on two MRI-based stroke lesion datasets to confirm the superior performance of the TransRender.

## 2. Related work

We will review the relevant literature from CNN-based methods, hybrid architecture-based methods, and boundary-related methods in this section.

### 2.1. CNN-based methods

In several fields, such as image classification and image segmentation, the CNN methods have gained enormous success (Zhao et al., 2021, 2023; Guo et al., 2022). Traditional segmentation methods generally use convolutional and pooling layers to extract local features and thus perform segmentation (Li et al., 2021). U-Net (Ronneberger et al., 2015) is a popular symmetric structure based on convolution layers. The skip connection serves as a bridge to connect different semantic information, making U-Net suitable for medical image processing tasks. Some studies on stroke attempt to improve the U-Net method to realize accurate lesion segmentation. D-UNet (Zhou Y. et al., 2021) utilizes the dimensional transformation module to extract the spatial information between slices through the combination of 2D detail features and 3D spatial features. The multi-inputs UNet (Zhang et al., 2021b) takes 3D diffeomorphic registration with the original MRI as inputs, providing rich prior knowledge for the subsequent UNet segmentation network. The CNN-based encoder is limited by convolutional operations and still lacks the ability to extract global information. Yang et al. (2019) proposed a network that adopts DenseUNet as the encoder and uses a long short-term memory module to fuse contextual information on the decoder. The two-stage U-Net (Agnes et al., 2022) proposes a feature combination module to efficiently extract global information. Unfortunately, these methods introduce global features from different perspectives, but do not qualitatively eliminate the limitations of the convolutional inherent receptive fields.

### 2.2. Hybrid architecture-based methods

Transformer has spread from NLP to computer vision since it is excellent at attracting long-distance information and encoding shape representations (Han et al., 2022). The vision transformer (ViT) (Dosovitskiy et al., 2021) is the first structure to be used for image classification tasks and obtains results that exceed the CNN methods. As the interest grows, ViT and its derived methods (Liu et al., 2021) display powerful performance in a series of visual segmentation tasks. Because of the complex structure and tissue intensity similarity of medical images, a pure transformer is hard to realize the desired segmentation outcomes. The hybrid architectures of CNN combined with transformer have become the model of choice in the medical field (He et al., 2022). TransUNet (Chen et al., 2021) is the first hybrid structure that is utilized to segment the abdominal organs. TransUNet extracts deep-level features by using stacked convolutional layers and then establishes long-term associations by stacking transformer layers in a cascade way. On the contrary, BiFusion module (Zhang et al., 2021a) is proposed to integrate the parallel convolutional and transformer branches, and the proposed method achieves excellent performance while being highly efficient. Swin-Unet is proposed by Cao et al. (2023), combining a Swin transformer with a U-shaped structure. Swin-Unet can capture local semantic features and build long-distance context information. The nnFormer is proposed by Zhou H. Y. et al. (2021), which optimally combines convolution with a self-attentive mechanism to surpass previous methods on brain tumor segmentation. As for the decoder, both of them employ the traditional convolutional upsampling path or transformer layers, which tend to degrade the boundary information due to the uniform computation of the pixels around the edge (Kirillov et al., 2020).

### 2.3. Boundary-related methods

We notice recent works in medical image segmentation that can be related to the proposed method. de Vries et al. (2023) adopts general architecture as the encoder-decoder, while they introduce the multiple cross-attention module to receive the temporal information. Zhu et al. (2023) proposed a fusion network that extracts edge features from CNN and edge spatial attention blocks, and fuses edge features with semantic features from the transformer. To clarify the structure boundaries, the boundary preserving module (Lee et al., 2020) is proposed to generate a key point map and explore the boundaries of the target object. Kirillov et al. (2020) proposed a unique idea of considering image segmentation as a rendering issue. The rendering-based approach is effective and qualitative in the instance segmentation and semantic segmentation tasks. In the boundary-rendering network (Huang R. et al., 2022), a point selection module is proposed to concentrate on the area of unclear edge. Moreover, a boundary rendering module is employed to discover the contour information. Some other methods (Chu et al., 2020; Kervadec et al., 2021) to design boundary loss functions to mitigate the difficulties of highly unbalanced problems in medical images. However, the existing methods tend to generate over-smooth or inaccurate predictions (Huang R. et al., 2022). We propose an improved render-based decoder and combine it with a transformer-based encoder, which can accurately segment lesions via fine-level details on a grid and global semantic information.

## 3. Methodology

**Figure 2:**
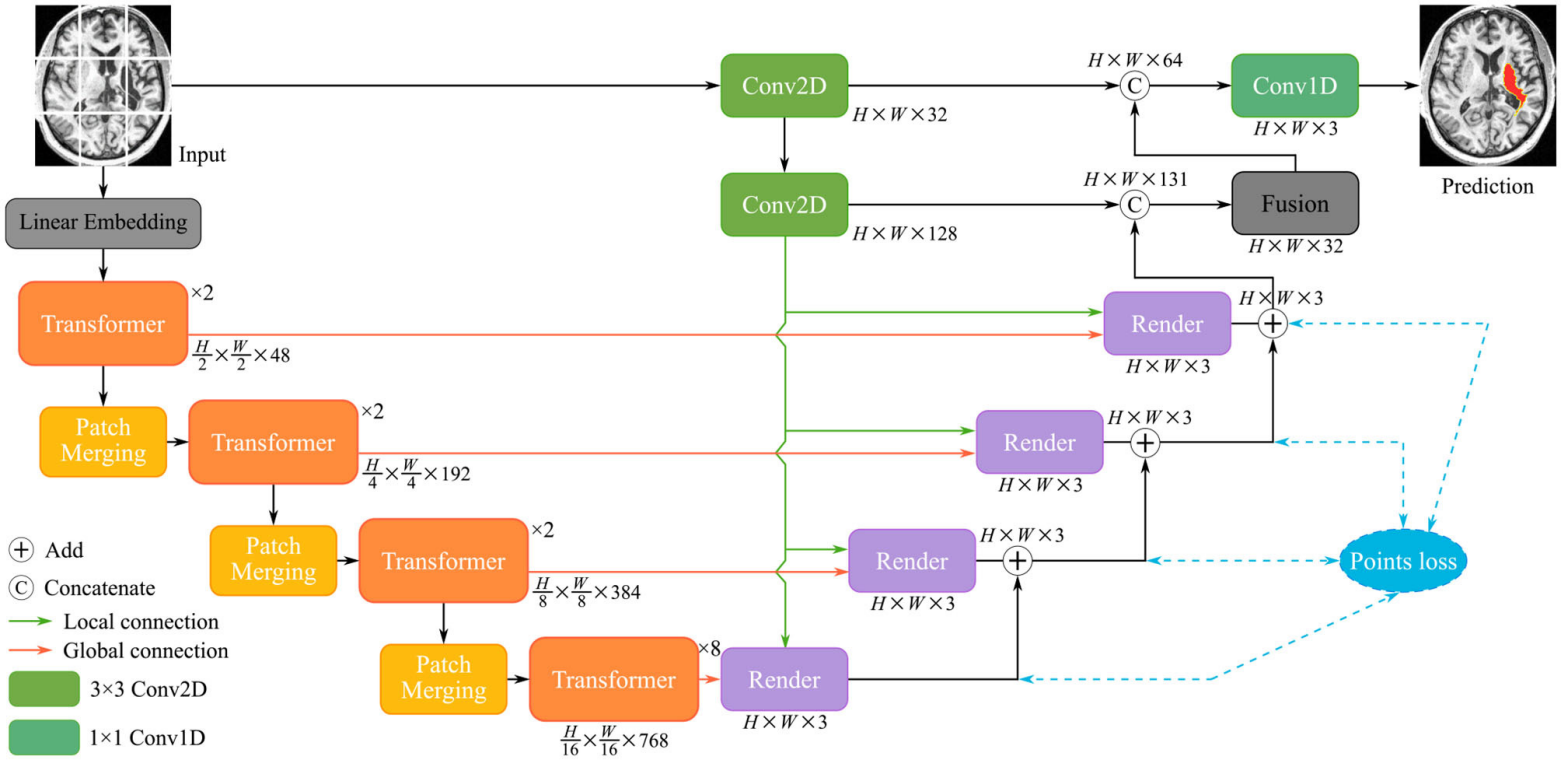
The structure of the TransRender, which includes a multi-scale transformer-based encoder, render-based decoder, and a fusion module. Moreover, the orange line, green line, and blue line mean global skip connection, local skip connection, and point-based loss function, respectively.

The structure of the TransRender is described in Figure 2. The transformer-based encoder, render-based decoder, and fusion module are the three parts of the proposed network architecture. For each sliced input image, TransRender utilizes a multi-scale transformer as an encoder to establish long-range dependencies between the patch sequences. Then, the render-based decoder recovers the resolution of the segmentation by upsampling strategy with local-global features. Finally, a fusion module is adopted as the postprocessing to integrate the segmentation maps at each level. Furthermore, the proposed method trains renders with several point-based supervisions. We introduce the detailed structure of these three parts in this section.

### 3.1. The encoder

Figure 2 gives the structure of the encoder, which mainly consists of several transformer modules and convolutional modules. To encode the hierarchical context information of the input image, we first utilize a hierarchical transformer. With a particular input *X*∈ℝ^*H*×*W*×*C*^, we denote its spatial resolution by *H*×*W* and its channel number by *C*, respectively. The MRI image *X* is initially split into a patch sequence $\left\{x_{p}^{i}\in {\mathbb{R}}^{P^{2}\cdot C}|i=1,..,N\right\}$ in the linear embedding layer, where the height and width of each patch are *P*_*H*_ and *P*_*W*_, and *N* stands for the amount of patches. Then we flatten and reflect these patches to a D-dimensional feature representation via the linear projection:


(1) $$z_{0}=[x_{p}^{1}E;x_{p}^{2}E;...;x_{p}^{N}E],\text{s}.\text{t}.z_{0}\in {\mathbb{R}}^{N\times D},E\in {\mathbb{R}}^{(P^{2}\cdot C)\times D},$$

where *z*_0_ represents the final features, and *E* is the patch embedding projection.

**Figure 3:**
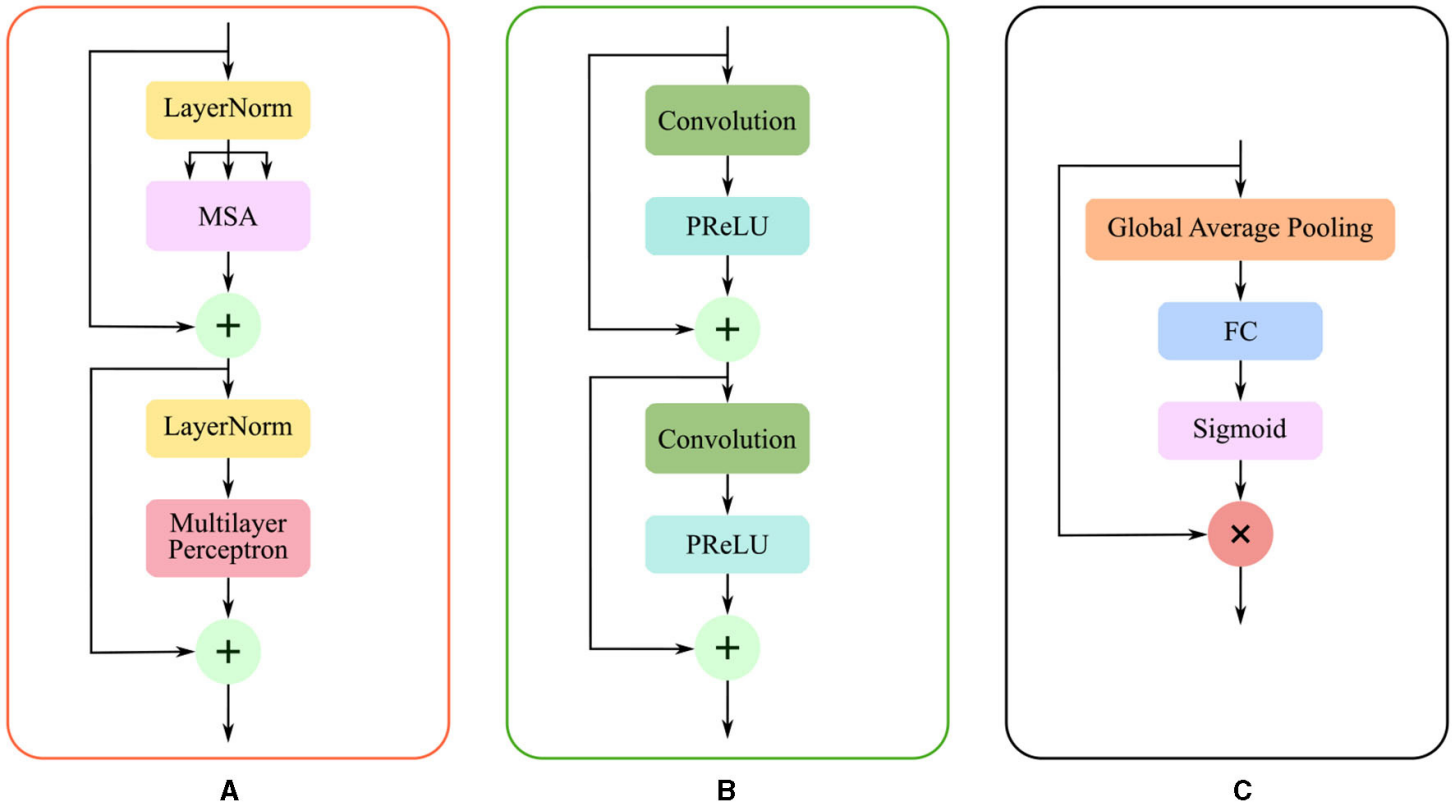
The structure of three modules: **(A)** Transformer layer composed of MSA, layer normalization, and MLP; **(B)** Conv2D module consist of two stacked of convolutional layers and activation functions; **(C)** Fusion module comprises GAP, FC layer, and *sigmoid* function.

Finally, a positional embedding $E_{pos}\in {\mathbb{R}}^{N\times D}$ to be added is significant for the divided patches to integrate positional information. The encoded patch sequence will be fed into the transformer layers. As illustrated in Figure 3A, the cascaded multi-head self-attention (MSA) layer and the multi-layer perception (MLP) layer comprise the transformer, which is computed as:


(2) $${t^{\prime }}_{l}=\text{MSA}(LN(t_{l-1}))+t_{l-1},$$

(3) $$t_{l}=\text{MLP}(LN({t^{\prime }}_{l}))+{t^{\prime }}_{l},$$

where *t*_*l*_ and *t*_*l*−1_ represents the resulting features of the corresponding transformer layers, and *LN*(·) denotes the layer normalization. The MSA is defined as:


(4) $$\begin{array}{l} {\text{MSA}}_{i}\left(X\right)=\sigma_{1}\left(\frac{Q_{i}{K_{i}}^{T}}{\sqrt{d}}\right)V_{i},\text{s.t.}Q_{i}=XW_{i}^{Q},K_{i}=XW_{i}^{K},V_{i}=XW_{i}^{V}, \end{array}$$

where *d* denotes the feature dimension, and $Q_{i}\in {\mathbb{R}}^{N\times D_{q}}$, $K_{i}\in {\mathbb{R}}^{N\times D_{k}}$, and $V_{i}\in {\mathbb{R}}^{N\times D_{v}}$ are the query, key, and value, respectively. The $W_{i}^{Q}$,$W_{i}^{K}$, and $W_{i}^{V}$ are the weight matrices, and σ_1_ means the nonlinear function *softmax*. Moreover, patch merging is employed between the two transformer modules, which reduces the spatial resolution of patches and doubles the channel dimension simultaneously.

As mentioned in the previous section, pure transformer architecture is not optimal for the different segmentation tasks. We utilize the convolutional modules additionally to enrich the local representation. In the initial stage of encoding, the undivided input *X*∈ℝ^*H*×*W*×*C*^ is directly fed into the Conv2D module. The structure of the Conv2D is shown in Figure 3B, which is defined as follows:


(5) $$\begin{array}{l} \text{Conv2D}(X)=\sigma_{2}(BN(C_{3}^{1}(\sigma_{2}(BN(C_{3}^{1}(X)))+X)))+\\ \;\sigma_{2}(BN(C_{3}^{1}(X)))+X, \end{array}$$

where $C_{3}^{1}\left(\cdot \right)$ denotes a two-dimensional convolution with the 3 × 3 kernel and the 1 × 1 stride, σ_2_ means the PReLU linear function, and *BN*(·) represents the batch normalization. TransRender extracts the local features and long-distance dependency of the image at the encoding stage, which will be used by the decoder to perform resolution recovery of the predicted image.

### 3.2. The decoder

**Figure 4:**
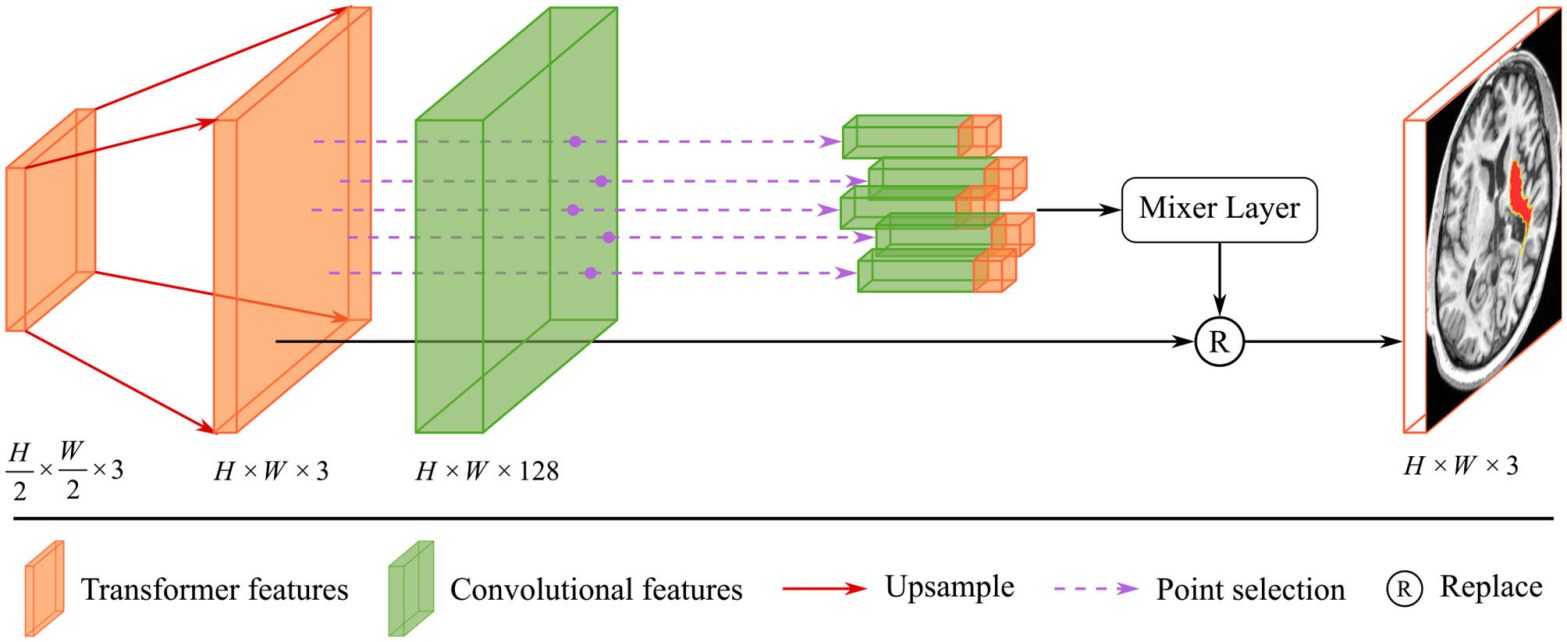
The structure of render module. With the transformer features from the global skip connection and convolutional features from the local skip connection as input, render adaptively selects key points by point selection strategy and combines point features to make re-prediction.

Due to the complexity of cerebral structures, the boundaries of stroke lesions are difficult to identify. The traditional CNN methods treat all pixels of the irregular target object uniformly in a convolutional way (Kirillov et al., 2020), either at the lesion boundary or the lesion core. And the proposed render module first selects the set of uncertain points and extracts the feature representations corresponding to these points, and implements the re-prediction of these uncertain points by using the prediction head. The accurate localization of the lesion boundary is accomplished by further prediction of the selected uncertainty points. We take several renders to build a decoder that adaptively predicts points with high uncertainty. The render mainly includes three steps, as shown in Figure 4: point selection strategy, point re-prediction, and point replacement.

**Figure 5:**
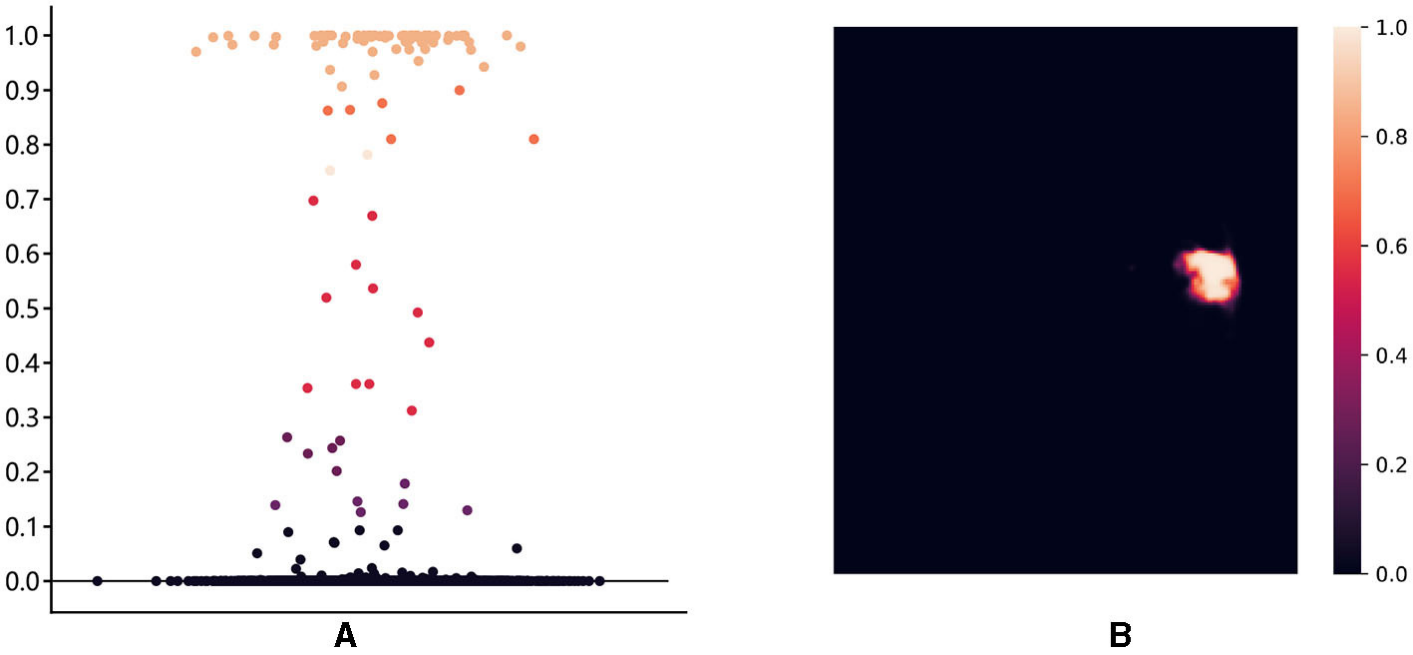
The distribution of pixel values and pixel locations: **(A)** The distribution of pixel points in different intervals, with [0.3, 0.7] as the high uncertainty interval; **(B)** The ambiguous pixel points are mainly distributed at the boundary of the lesion core area.

First, we introduce the point selection strategy using the last layer of the proposed method as an example. For the given feature map $X_{t}\in {\mathbb{R}}^{\frac{H}{2}\times \frac{W}{2}\times 1}$, the render first upsamples it by a 2 × interpolate function to obtain the initial coarse segmentation ${\hat{X}}_{t}\in {\mathbb{R}}^{H\times W\times 1}$. The values from [0, 1] on the segmentation ${\hat{X}}_{t}$ represent the possibility of whether the current pixel is a lesion or not. We give the distribution of pixel values and pixel positions in Figure 5. Different colors of points represent different values, where black, orange, and red represent healthy tissue, lesions, and fuzzy boundaries, respectively. If the value of the pixel is closer to 0, it is more likely that the current pixel is a background (healthy tissue), and vice versa, if the value is closer to 1, it means a lesion. When the segmentation threshold is set to 0.5, the closer the threshold is, the higher the uncertainty. Although the number of pixel points near the threshold is sparse in Figure 5A, they are essential for the clear localization of the boundary. These values are sorted in descending order for each pixel, which is calculated as follows:


(6) $$\begin{array}{l} \forall 1\leq h\leq H,\forall 1\leq w\leq W,M_{s}=\left\{p_{h,w}^{1},p_{h,w}^{2},..,p_{h,w}^{n}\right\}, \end{array}$$

where $p_{h,w}^{i}$ represents the prediction value at the (*h, w*) location and *n* = *H*×*W* denotes the number of pixels, *M*_*s*_ is the feature map derived after sorting, and the point in the *M*_*s*_ follows the rule that $p_{h,w}^{i}$ is greater than $p_{h,w}^{i+1}$. Based on the *M*_*s*_, we construct the uncertain points map *M*_*u*_. It can be obtained as follows:


(7) $$\begin{array}{l} M_{u}=|p_{h,w}^{i}-p_{thd}|, \end{array}$$

where *p*_*thd*_ represents the threshold value of uncertainty. For the final uncertainty map *M*_*u*_, a smaller value at a pixel means that the segmentation network has more uncertainty in the prediction. That means the smaller the difference between $p_{h,w}^{i}$ and *p*_*thd*_, the larger the uncertainty of the current pixel. To eliminate the strong bias due to the space position, the proposed render sampling *k*×*N* points across the *M*_*u*_ at random. It identifies that the β × *N* points with the highest uncertainty in the set of points are located around the boundary region, as shown in Figure 5B. These points will be important to correct the segmentation at the lesion boundary.

Then, the render module integrates the features based on the selected points, combining the contextual semantic information from the global skip connection and local detail information from the local skip connection. The feature sequences corresponding to the selected points are fed into the mixer layer for point-based re-prediction, where the mixer layer consists of two trainable MLP layers. Finally, the re-predicted points set replaces the high uncertainty points set in the initial segmentation to accomplish the precise localization of the lesion boundary. The structure of the render is shown in Figure 4.

Based on the render module proposed above, we construct the render-based decoder (see in Figure 2), which combines local and global features at multiple scales. Furthermore, we introduce a fusion module at the end of the decoder in Figure 3C, which fuses multiple layers of decoded features. The segmentation of the different levels renders is merged as input *X*_*r*_ to perform the following operations:


(8) $$\begin{array}{l} A\left(X_{r}\right)=\sigma_{3}\left(FC\left(GAP\left(X_{r}\right)\right)\right)+X_{r}, \end{array}$$

where *GAP*(·), *FC*(·), and σ_3_ denotes global average pooling, fully connected layer, and *sigmoid* function, respectively. The fusion module emphasizes segmentation-related information and suppresses irrelevant features in an attentive manner.

### 3.3. Loss function

The multi-scale render decoder adaptively selects the boundary key points, thus improving the segmentation performance. In the training stage, we design a combined loss function from two aspects: segmentation loss and point loss, which is calculated as:


(9) $${ℒ}_{total}(p,g)={ℒ}_{dice}(p,g)+\lambda \sum_{i=1}^{n}{ℒ}_{bce}^{i}(p,g),$$

where ℒ_*dice*_(*p, g*) indicates segmentation loss and ℒ_*bce*_(*p, g*) is point loss. λ represents the weight parameter, and the default setting is λ = 0.7. The segmentation loss supervises the network to generate regional details in the whole upsampling recovery process, and point-to-point losses are employed to monitor each render module in the decoder. The weight parameters of the MLP layer in render are dynamically updated when the point selection strategy calculates the point loss between the selected points on ground truth and the points after re-prediction. Both two loss functions are calculated as:


(10) $${ℒ}_{dice}(p,g)=1-\frac{2\sum_{i=1}^{N}p_{i}g_{i}+\delta }{\sum_{i=1}^{N}p_{i}^{2}+\sum_{i=1}^{N}g_{i}^{2}+\delta },$$

(11) $${ℒ}_{bce}(p,g)=-(p\log(g)+p^{\prime }\log(g^{\prime })),$$

where *p* represents the prediction probability, *g* represents the expert annotation. *p*′ and *g*′ represents the contrary prediction probability of *p* and *g*, respectively.

## 4. Experiments and configurations

### 4.1. Datasets

Different stroke lesion segmentation datasets, including 490 MRI images, are used to conduct experiments to validate the proposed method. They include brain MRIs of stroke patients in the acute, sub-acute, and post-stroke stages. The details of both datasets are introduced as follows.

The anatomical tracings of lesions after stroke (ATLAS) is a publicly available dataset that includes 240 MRI images. Each image contains the MRI for the t1-weighted modality and the corresponding lesion annotation. The ischemic stroke lesion segmentation (ISLES2022) is provided for use at the MICCAI 2022 grand challenge, which contains 250 MRI images. In contrast to ATLAS, ISLES2022 contains three different modalities: ADC, DWI, and FLAIR. The original size of ATLAS is 233 × 197 × 189, while the original size of ISLES2022 varies over a wide range. After we slice these 3D MRIs into 2D images, the slices are resized to a uniform resolution of 208 × 176. In Table 1, we compare both two datasets in terms of imaging method, data source, modality, number of images, and dataset division.

**Table 1:** The data comparison of ATLAS and ISLES2022 dataset.

**Dataset**	**Imaging method**	**Data source**	**Modality**	**Number of images**	**Dataset division**
ATLAS	MRI	Public	T1WI	240	160/40/40
ISLES2022	MRI	Public	DWI, ADC, FLAIR	250	168/41/41

### 4.2. Configurations

The PyTorch framework and Python are used to carry out the experiments. We adopted AdamW as the optimizer with default parameter settings. The epoch-based early stop strategy is utilized to determine whether the model optimization is complete. Furthermore, the transformer layers are pre-trained on the large images dataset. All experiments are performed on GeForce RTX 2080 super with 8 GB memory.

We select common metrics to measure the advantages of TransRender, including DSC, Precision, Recall, and HD to evaluate the similarity between prediction results and lesion labels. We consider the first two metrics, DSC and HD, more significant than the classic F2, Precision, and Recall. The DSC calculates the region similarity, and the HD calculates the boundary similarity between the two inputs.

### 4.3. Experiments

#### 4.3.1. Comparison experiment

We compare our TransRender with previous methods: U-Net (Ronneberger et al., 2015), AG U-Net (Schlemper et al., 2019), D-UNet (Zhou Y. et al., 2021), CLCI-Net (Yang et al., 2019), SAN-Net (Yu et al., 2023), TransUNet (Chen et al., 2021), TransFuse (Zhang et al., 2021a), and MLRA-Net (Wu et al., 2022) using ATLAS dataset to illustrate efficiency of the TransRender. Table 2 shows the performance comparison, where the experimental result of the proposed TransRender is presented in the last line. Further experiments are implemented on the ISLES2022 to validate the generalizability of the TransRender, as shown in Table 3. All of the above experiments employ cross-validation methods to avoid randomness.

**Table 2:** The quantitative comparison of TransRender with the previous eight methods on the ATLAS dataset.

**Method**	**DSC (F1) (*%*)**	**HD (*px*)**	**F2 (*%*)**	**Precision (%)**	**Recall (%)**
U-Net (Ronneberger et al., 2015)	48.34	51.35	49.50	54.45	53.68
AG U-Net (Schlemper et al., 2019)	49.60	50.12	53.67	49.25	62.53
CLCI-Net (Yang et al., 2019)	51.74	–	51.28	–	51.39
MI-Net (Zhang et al., 2021b)	56.72	38.80	–	60.90	59.38
SAN-Net (Yu et al., 2023)	57.11	–	56.23	–	59.77
D-UNet (Zhou Y. et al., 2021)	53.49	–	–	63.31	52.43
TransUNet (Chen et al., 2021)	56.23	45.44	59.64	57.15	65.95
TransFuse (Zhang et al., 2021a)	58.18	41.56	**62.40**	57.64	**70.06**
TransRender	**59.79**	**33.98**	59.38	**63.91**	68.08

The bold values in the table represent the best results.

**Table 3:** The performance comparison of TransRender with the previous five methods on the ISLES2022 dataset.

**Method**	**DSC (F1) (%)**	**HD (px)**	**F2 (%)**	**Precision (%)**	**Recall (%)**
U-Net (Ronneberger et al., 2015)	82.04	36.82	81.52	85.31	81.44
AG U-Net (Schlemper et al., 2019)	81.45	37.01	80.99	84.70	80.98
TransUNet (Chen et al., 2021)	84.23	29.98	84.01	86.88	84.19
TransFuse (Zhang et al., 2021a)	84.39	29.19	84.06	**87.36**	84.15
MLRA-Net (Wu et al., 2022)	84.73	29.95	84.48	87.03	**84.70**
TransRender	**85.37**	**27.60**	**84.87**	86.48	83.94

The bold values in the table represent the best results.

#### 4.3.2. Ablation experiment

The four ablation experiments on decoders are conducted to assess the availability of the render module, which are shown below: (1) The encoder uses U-Net and traditional convolutional upsampling path as the decoder; (2) The encoder uses U-Net and render-based upsampling path as the decoder; (3) TransRender as the encoder and traditional convolutional upsampling path as the decoder; (4) TransRender as the encoder and render-based upsampling path as the decoder. Table 4 shows the comparative results.

**Table 4:** The ablation comparison of TransRender on the ATLAS dataset.

	**Encoder**	**Render**	**DSC (F1) (%)**	**HD (px)**
CNN	U-Net	–	48.34	51.35
		✓	54.13	40.71
	AG U-Net	–	49.60	50.12
		✓	**55.21**	**38.14**
Transformer	TransUNet	–	56.23	45.44
		✓	57.86	37.42
	TransRender	–	58.27	37.86
		✓	**59.79**	**33.98**

The bold values in the table represent the best results.

#### 4.3.3. Hyper-parameter comparison

The render module automatically selects *k*×*N* points as the uncertain points set to predict. The value of *k* directly affects how many points are selected in network learning and, consequently, the segmentation capacity of the proposed TransRender. We set *k* = 1, 2, 3, 5 in the render module to compare the performance using ATLAS dataset, respectively. Table 6 shows the results of this experiment. It is also worthwhile to investigate the value of β, which indicates the different percentiles of points selected as important points. These important β × *N* points are sampled for the features of spatial location, while the other (1−β) × *N* points are randomly assigned features. We conduct a comparative experiment to explore the effect on segmentation performance by using β = 0.1, 0.5, 0.6, 0.7, 0.8 on the ATLAS dataset, respectively. The results of this experiment as shown in Table 7.

## 5. Result and discussion

### 5.1. Comparison experiment

Table 2 reports the quantitative results using the ATLAS dataset. Comparative experiments with eight different existing methods are conducted to analyze the segmentation effectiveness of the point-based TransRender. The comparison results indicate that TransRender exceeds the previous method, performance gains range from 1.61%, 7.58*px*, and 0.60–11.45%, 17.37*px*, and 9.46% considering the DSC, HD, and PRE, respectively. The significant improvements demonstrate that applying a render-based decoder to TransRender is better at capturing boundary semantic information than a standard decoder. For the DSC, our method achieves a mean DSC of 59.79%, which is improved by 2.77% than the second-best TransFuse. We would also like to mention that the difference in the HD metric is pretty large. Our method does not obtain the best performance in terms of F2 and RE, only 59.38 and 68.08%, which are the third- and second-best ranks, respectively. However, we recognize that region overlap (DSC) and boundary distance (HD) is more important between the prediction results and the physicians annotation. Excellent results verify that adaptively predicting selected points can improve lesion segmentation at the boundary.

**Figure 6:**
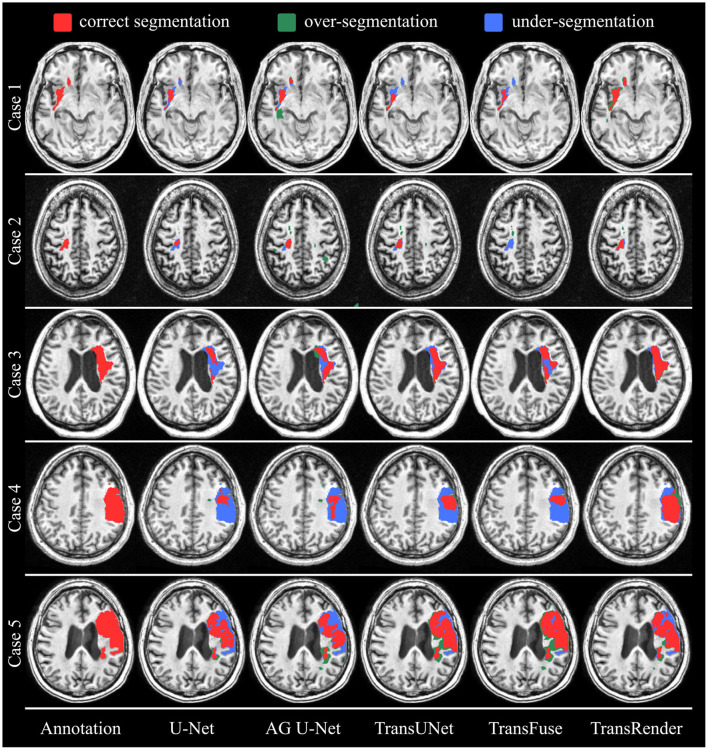
The visual segmentation results of TransRender and the four previous methods on the ATLAS dataset. Where, the red color, blue color, and green color mean correct, insufficient, and excessive segmentation, respectively.

Furthermore, the qualitative comparisons of the ATLAS dataset are displayed in Figure 5. As we can see from the visualization results, whether the lesion size is large or small, the lesion location is left or right, our method produces visually superior segmentation. We visualize four methods, including U-Net (Ronneberger et al., 2015), AG U-Net (Schlemper et al., 2019), TransUNet (Chen et al., 2021), and TransFuse (Zhang et al., 2021a) to compare visually with the TransRender. The scale, location, and shape of each lesion are different in the selected five brain images. In Case 1, the target object consists of an infarct lesion and multiple embolic, the latter of which size is extremely small. All methods identify infarct lesions with more or less accuracy, but our TransRender achieves the best regional similarity. And for the multiple embolic, only AG U-Net and TransRender locate the lesion, where the latter obtains more correct segmentation and less over-segmentation. The lesion size in Case 2 is small, so U-Net and TransFuse only segment a small part of the lesion or even fail to identify it. The other two previous methods realize correct segmentation almost completely, but at the cost of severe over-segmentation. Benefiting from the prediction of the boundary key points by the render module, the proposed method greatly reduces over-segmentation. In Case 3, transformer-based methods display significantly improved segmentation performance compared to CNN-based methods. However, these methods suffer from different degrees of under-segmentation. The TransRender yields precise details of the lesion boundary, with almost no under-segmentation. We regard Case 4 in Figure 6 as a difficult segmentation issue due to its close location to the skull. None of the five methods completely segments the lesion, whereas TransRender achieves the correct segmentation of the most pixels. It is necessary to mention that TransRender suffers from a slight over-segmentation. The complex tissue structure in the area of the focal lesion affects the segmentation performance of all methods. So in Case 5, the prediction results of each method are coarse and discontinuous. TransRender yields fewer over-segmentation than the transformer-based methods, and fewer under-segmentation than the CNN-based methods.

We further carry out comparisons to validate the performance robustness of the TransRender. The quantitative comparison is reported in Table 3 between the TransRender and five methods using the ISLES2022 dataset. We can observe that the CNN methods are significantly worse than that the transformer methods in terms of five metrics. MLRA-Net outperforms fourth-best TransUNet and third-best TransFuse by 0.50 and 0.34% on the DSC metric, respectively, but it is worse than TransFuse on the HD metric. The proposed method uses a multi-scale transformer as the encoder with render as the decoder that yields the best scores on the DSC, HD, and F2 metrics. It might be interpreted that render successfully corrects the error segmentation at the lesion boundary.

**Figure 7:**
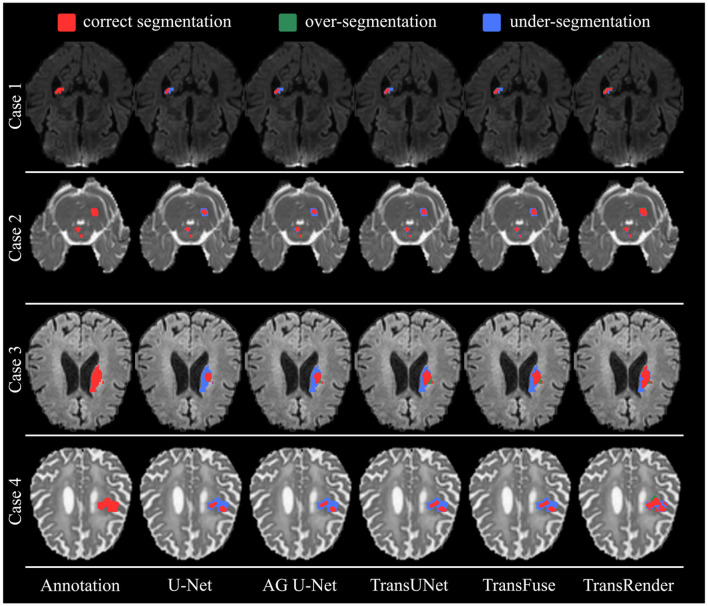
The visual segmentation results of TransRender and the four previous methods on the ISLES2022 dataset. Where, the red color, blue color, and green color mean correct, insufficient, and excessive segmentation, respectively.

Figure 7 displays the qualitative comparison using the ISLES2022 dataset. Four brain images are selected for visualization and comparison, each of which has different modalities, lesion shapes, and locations. In Case 1 and Case 3, all methods only segment parts of the lesion to a more or less degree, while TransRender realizes the best region overlap and boundary similarity. The lesions in Case 2 are multiple embolic, and only the proposed method segments the lesions nearly completely. The excellent results on these two datasets validate the segmentation accuracy of the TransRender for multiple embolic. The existing methods all identify Case 4 as having multiple lesions, and the reason may be that the lesion occurs in the cerebral cortex. TransRender identifies Case 4 as a whole lesion and completes more correct segmentation.

Overall, on these two datasets, the proposed TransRender can yield satisfactory segmentation performance, both qualitatively and quantitatively. These results indicate the efficacy and robustness of TransRender for stroke lesion segmentation.

### 5.2. Ablation experiment

Ablation experiments on decoders are conducted to investigate the impact of the render module on lesion segmentation. The comparison results for performance and complexity are presented in Tables 4, 5. When using the render-based decoder, the DSC scores of both U-Net and TransRender are improved, while the HD scores are descended. We carry out experiments with the U-Net or TransRender as encoders, and the classical convolutional upsampling or render modules as decoders, respectively. The DSC and HD using a convolutional upsampling- and render-based decoder are improved from 48.34% and 51.35*px* to 54.13% and 40.71*px*, which gain improvements of 11.98 and 20.72%. With TransRender as the encoder, we employ render as the decoder, which attains superior performance, scoring 59.79% in DSC and 33.98*px* in HD. It is worth noting that by using render as the decoder, the calculation complexity and the network parameters are also decreased. These ablation comparisons demonstrate that the proposed render offers a competitive advantage over convolution methods in terms of its ability to process high-frequency information at the boundary.

**Table 5:** The complexity comparison of TransRender and U-Net w/o Render.

**Encoder**	**Render**	**FLOPs (*G*)**	**Params (*M*)**
U-Net	–	30.5	31.0
	✓	**15.6**	**18.9**
TransRender	–	118.4	43.6
	✓	**100.1**	**32.2**

The bold values in the table represent the best results.

### 5.3. Hyper-parameter comparison

Further comparison experiments are conducted to explore whether the hyper-parameters *k* and β would affect the segmentation performance. Table 6 presents the comparison results using different numbers of selected points. The number of points selected is desired to match the lesion due to the different sizes. When *k* = 3, TransRender gives the best result in all metrics. In the experiments, we set *k* = 3 by default. The performance comparison using the different numbers of important points is shown in Table 7. The comparison indicates that there is a significant influence of β values on the segmentation. Note that we set β = 0.1 to suppress the features of important points and highlight the random features of other points. The comparison results indicate that more important points should be selected for feature extraction. As β increases, the segmentation performance becomes more favorable until β = 0.7. This might be due to some point features that mistakenly guide the decoding process. In the other experiments, we set β to 0.7 by default.

**Table 6:** Segmentation performance comparison of different initial *k*.

**Value of *k***	**DSC (F1) (*%*)**	**HD (*px*)**	**F2 (*%*)**
1	59.07	34.96	58.97
2	59.27	34.77	59.21
3	**59.79**	**33.98**	**59.38**
5	59.26	34.85	59.17

The bold values in the table represent the best results.

**Table 7:** Segmentation performance comparison of different β.

**Value of β**	**DSC (F1) (*%*)**	**HD (*px*)**	**F2 (*%*)**
0.1	55.17	45.33	54.99
0.5	58.76	38.49	57.48
0.6	59.38	36.90	**59.61**
0.7	**59.79**	**33.98**	59.38
0.8	59.02	34.57	57.24

The bold values in the table represent the best results.

## 6. Conclusion

In this study, we propose a novel point-based boundary segmentation method for stroke lesions using different MRI images. The TransRender is built on a multi-scale transformer encoder because of its strong ability to establish long-distance dependencies. The render-based decoder implements the non-uniform grid representation, which allows more attention to the precise features at the boundaries. Furthermore, a combined supervision loss is utilized to optimize the point selection of the render. Extensive experiments are conducted using the different ischemic stroke datasets to evaluate TransRender. And the experimental results indicate that TransRender has a competitive advantage over the existing networks in terms of both accuracy and complexity. Unfortunately, the improved render module is not adequate to achieve accurate segmentation due to the variety of lesions. We may consider the use of other network structures in the future to accomplish the re-prediction of selection points in the render module.

## Data availability statement

The original contributions presented in the study are included in the article/supplementary material, further inquiries can be directed to the corresponding authors.

## Author contributions

ZW: Conceptualization, Methodology, Visualization, Writing—original draft. XZ: Software, Supervision, Writing—review and editing. FL: Investigation, Supervision, Writing—review and editing. SW: Formal analysis, Validation, Writing—review and editing. JL: Validation, Writing—original draft, Writing—review and editing.
